# Technical performance scores associate with early prognosis of tetralogy of Fallot repair

**DOI:** 10.3389/fped.2024.1274913

**Published:** 2024-01-31

**Authors:** Ailixiati Alifu, Haifan Wang, Renwei Chen

**Affiliations:** Department of Cardiothoracic Surgery, Hainan Women and Children’s Medical Center, Haikou, Hainan, China

**Keywords:** technical performance score, tetralogy of Fallot, early prognosis, congenital heart disease, reintervention

## Abstract

**Objective:**

This study aimed to investigate the relationship between technical performance scores (TPS) and the early prognosis of tetralogy of Fallot repair (TOF).

**Methods:**

A retrospective study was conducted on TOF repair patients at our center from Oct 2017 to Oct 2022. Patients were classified into Class 1 (no residua), Class 2 (minor residua), or Class 3 (major residua) based on TPS derived from predischarge echocardiograms and need for reintervention. Statistical methods were used to assess the association between TPS and early prognosis.

**Results:**

A total of 75 TOF repair patients (40% female, 60% male) were analyzed and categorized into TPS1 (24%), TPS2 (53.3%), and TPS3 (22.6%) based on pre-discharge echocardiographic findings. The median follow-up time was 7.0 months. The multivariable Cox regression analysis indicated that TPS3 scores are associated with a 12.68-fold increase in risk compared to TPS1 and TPS2 scores [95% CI = 12.68 (0.9∼179.28), *P* = 0.06]. The Spearman rank correlation analysis revealed a weak positive correlation between TPS classification and low cardiac output syndrome (*r* = 0.26, *P* = 0.03). However, there were no significant differences in ICU stay or duration of mechanical ventilation among the groups.

**Conclusion:**

TPS3 after intracardiac TOF repair is associated with higher risk of early re-intervention, highlighting the importance of close follow-up and monitoring in this patient population. Patients who develop low cardiac output syndrome in the early postoperative period may have residual defects that require prompt identification.

## Introduction

TOF is the most common cyanotic congenital heart disease (CHD), with an incidence of four cases per 10,000 live births (1). The first report of a corrective surgical procedure for TOF was in 1954, where 106 patients underwent surgery with a 30-year survival rate of 77% (2). Surgical repair has evolved from initially completely relieving right ventricular outflow tract obstruction, which caused pulmonary valve regurgitation, to the current strategy of preserving the valve and allowing mild pulmonary valve stenosis (3). The TPS was initially developed by Larrazabal et al. for common surgical procedures in CHD and is a tool for assessing the adequacy of correction of cardiac anomalies (4). Multiple studies have demonstrated that TPS grading is correlated with early and long-term prognosis after common CHD surgeries (5–8). Based on echocardiography and clinical standards, the operative procedures for CHD are subdivided into sub-operations, and TPS is graded as 1 (no residual), 2 (minor residua), or 3 (major residua, pacemaker implantation or reintervention for major residua prior to discharge). If all sub-operations are graded as 1, the total score for the entire operation is 1 (optimal). If the highest score for any sub-operation is 2, the TPS for the entire operation is 2 (sufficient). If any sub-operation is graded as 3, the TPS for the entire operation is 3 (insufficient) (4). We hypothesized that TPS associate with early prognosis of tetralogy of Fallot repair.

## Patients and methods

This is a single-center, retrospective cohort study, which has been approved by the Institutional Review Board (IRB) of Hainan women and children's medical center [LL00254]. Written informed consent was waived by the Institutional Review Board (IRB) of Hainan women and children's medical center due to the anonymous nature of the retrospective study. All the methods were carried out in accordance with the Helsinki Declaration guidelines. We retrospectively reviewed 87 consecutive cases who underwent repair TOF from October 2017 to October 2022 at Hainan women and children's medical center. The exclusion criteria were as follows: TOF with pulmonary artery atresia, age greater than 2 years old, neonates, emergency surgery, and staged surgery.

### Surgical procedure

A median sternotomy was performed under low-temperature cardiopulmonary bypass with aortic root infusion of cold-blood cardioplegia for protection during TOF repair. The decision to incise the pulmonary valve annulus was based on preoperative echocardiography measurement of the *Z* value of the pulmonary valve annulus. If the *Z* value was less than −2.5, the pulmonary valve annulus was incised. If the *Z* value was between −2.5 and −2.0, the decision to incise the pulmonary valve annulus was made intraoperatively. If the *Z* value was greater than −2.0, the pulmonary valve annulus was not incised.

### Date collection

The data collected during hospitalization for all patients included age, gender, weight, height, coexisting anomalies, presence of chromosomal abnormalities, surgical approach, cardiopulmonary bypass (CPB) time, Aortic cross-clamp time, postoperative intensive care unit (ICU) stay, mechanical ventilation, occurrence of low cardiac output syndrome (LCOS), follow-up information after discharge, and perioperative discharge, and follow-up echocardiographic results.

### TPS grading

The TPS grading for postoperative TOF includes the following components: residual shunt in the atrial defect, residual shunt in the ventricular defect, residual shunt in the patent ductus arteriosus, adequate relief of right ventricular outflow tract obstruction, pulmonary valve stenosis or regurgitation, pressure gradient across the pulmonary artery trunk, pressure gradient across the left pulmonary artery, pressure gradient across the right pulmonary artery, and the occurrence of conduction block after surgery. Each component is assigned a score based on the following criteria: Grade 1 indicates no residual lesions or insignificant residuals; Grade 2 indicates minor residuals; Grade 3 indicates major residuals or the need for significant intervention. If all components are Grade 1, then the overall TPS grade is 1; if one or more components are Grade 2, then the overall TPS grade is 2; if any component is Grade 3, then the overall TPS grade is 3 (5, 9) (Supplementary Table S1).

### Definition of outcome

The primary outcome was a re-intervention within one year of discharge, which involved either a catheter intervention or surgery due to anatomical issues related to the initial repair of TOF.

LCOS was determined during the postoperative monitoring period by using Hand-held noninvasive cardiometer (ICON, Osypka Medical Germany). It was defined as a cardiac index (CI) of <2.0 L/(min m^2^).

### Statistical analysis

Data analysis was conducted using SPSS version 22.0, with visualizations generated through R software version 4.1.2. Categorical variables were presented as percentages and assessed using the Chi-squared test or Fisher's exact test as appropriate. Continuous variables adhering to a normal distribution were described using the mean ± standard deviation (SD). In contrast, those with a skewed distribution were reported as medians with interquartile ranges (IQRs) spanning the 25th–75th percentiles. To compare continuous data across three distinct groups, we employed the Kruskal–Wallis test. We calculated standardized mean differences (SMDs) to assess the consistency of baseline characteristics of baseline characteristics among different TPS groups. Variables exhibiting an SMD greater than 0.2 were considered imbalanced and were thus selected as covariates for adjustment in the subsequent multivariable Cox regression analysis. Correlation strength was quantified using Spearman's rank correlation coefficient, with the absolute value of r serving as the indicator: values from 0.7 to 1.0 signified a very strong correlation, 0.3 to 0.7 denoted a moderate correlation, and 0 to 0.3 pointed to a weak correlation (10). Survival outcomes were illustrated using Kaplan–Meier curves and compared with the Log-rank test. A 95% confidence interval was employed to estimate the precision of the survival rates. All statistical tests were two-sided, with a significance level set at *α* = 0.05.

## Results

87 patients underwent TOF repair, of which 12 were excluded (3 neonates, 4 patients over the age of 2 years, 3 patients who underwent staged surgery, and 2 lost to follow-up), leaving a total of 75 patients (86.2%) for inclusion in the study, with 60% male and 40% female. Based on pre-discharge echocardiography results, the patients were classified into TPS1 (18 cases, 24%), TPS2 (40 cases, 53.3%), and TPS3 (17 cases, 22.6%). The median follow-up time was 7.0 months. Statistically significant differences exist between the TPS1 and TPS3 groups in terms of height and transannular patch usage rate, as well as between the TPS2 and TPS3 groups regarding weight. However, no significant differences are observed among the three groups concerning age, gender, or congenital malformation rate (Table 1).

**Table 1:** Preoperative and intraoperative characteristics of different TPS.

Variation	TPS1 (*n* = 18)	TPS2 (*n* = 40)	TPS3 (*n* = 17)	TPS1 vs. TPS3	TPS2 vs. TPS3	TPS1 vs. TPS2	TPS1 and TPS2 vs. TPS3
				SMD			
Age, year [Median (IQR)]	1.04 (0.8, 1.6)	1.05 (0.5, 1.6)	1.24 (1.1, 1.4)	0.08	0.02	0.04	0.16
Gender, male, *n* (%)	11 (61.1)	25 (62.5)	9 (52.9)	0.17	0.20	0.03	0.18
Heigh, cm ((Mean ± SD)	72.3 ± 8.2	70.8 ± 12.2	74.7 ± 12.1	**0** **.** **28**	**0**.**32**	0.19	**0**.**28**
Weight, kg (Mean ± SD)	7.9 ± 1.7	7.1 ± 2.9	7.0 ± 1.9	**0**.**50**	0.01	**0**.**49**	0.14
Combined malformation (%)	3 (16.7)	4 (10.0)	3 (17.6)	0.03	**0**.**26**	0.18	0.15
Chromosomal abnormalities (%)	1 (5.6)	1 (2.5%)	2 (11.7)	0.01	**0**.**22**	0.13	0.10
CPB time, min [Median (IQR)]	88 (79.6,95.5)	92 (86,101.8)	95 (89,112.5)	**1**.**05**	**0**.**59**	**0**.**50**	**0**.**58**
Aortic cross-clamp time, min [Median (IQR)]	68 (61,73.3)	64 (54.8,71.0)	66 (57.0,75.0)	0.02	**0**.**29**	**0**.**36**	**0**.**22**
Used transannular patch, *n* (%)	13 (76.4)	27 (67.5)	9 (52.9)	**0**.**43**	**0**.**31**	0.11	**0**.**32**

Bold numbers indicate that SMD is greater than 0.2; SMD, standardized mean differences; TPS, technical performance scores; CPB, cardiopulmonary bypass.

**Figure 1:**
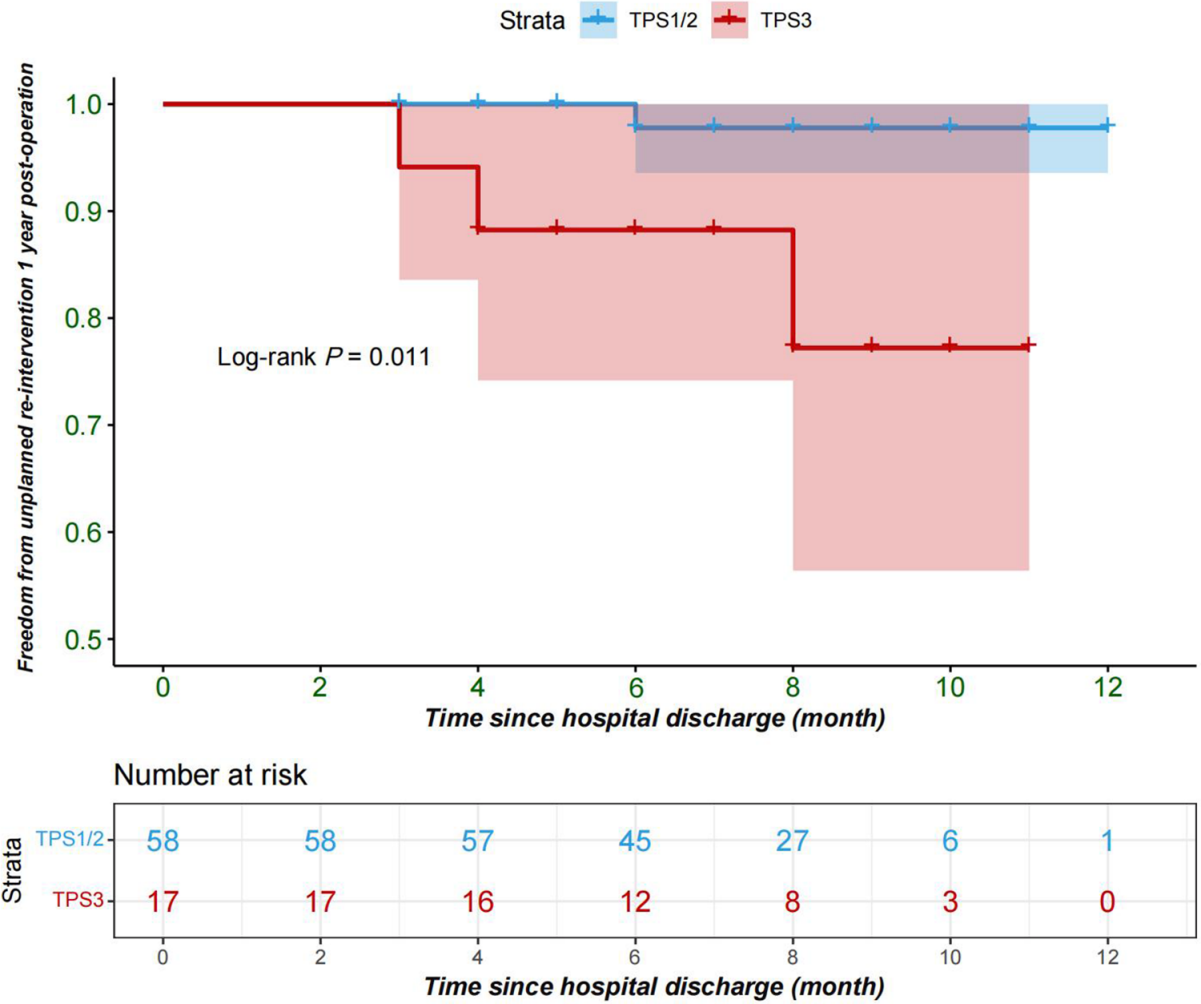
Freedom from re-intervention 1 year after repair of tetralogy of Fallot; TPS1, TPS2(blue); TPS3(red); TPS, technical performance score.

The primary outcome: The rate of reintervention within 1 year after discharge was 5.3% (4 cases) among the 75 patients included in the study, with no cases in TPS1, 1 case in TPS2, and 3 cases in TPS3. One case in TPS2 underwent catheter occlusion due to residual ventricular septal defect, while the two cases in TPS3 underwent catheter balloon dilation due to pulmonary valve stenosis, and one patient underwent catheter balloon dilation for left pulmonary artery stenosis. The Kaplan–Meier survival curve indicates that patients in the TPS3 group have a higher incidence of re-intervention within one year following the procedure, when compared to the TPS2 and TPS1 groups (log-rank *P* = 0.01) (Figure 1). The multivariable Cox regression analysis indicates an association in which TPS3 scores are associated with a 12.68-fold increase in risk compared to TPS1 and TPS2 scores [95% CI = 12.68 (0.9–179.28), *P* = 0.06]; however, this association is not statistically significant (Table 2).

**Table 2:** Multivariable Cox regression analysis of the association between various variables and the risk of re-intervention within one year.

Variable	HR (95% CI)	*P*
TPS3	12.68 (0.9–179.28)	0.06
age	1.14 (0.37–3.55)	0.82
sex (female)	0.12 (0–3.48)	0.22
Height	0.99 (0.88–1.12)	0.90
CPB time	0.98 (0.88–1.1)	0.78
Aortic cross-clamp time	1.09 (0.97–1.23)	0.13
Used transannular patch	0.18 (0.01–2.41)	0.20

There was no significant correlation between TPS classification and the secondary outcome indicators of ICU stay or postoperative mechanical ventilation time (*P* > 0.05). However, a statistically significant difference was observed in the incidence of postoperative LCOS among different TPS classifications (*P* < 0.05). (Table 3) Furthermore, The Spearman rank correlation analysis revealed a weak positive correlation between TPS classification and low cardiac output syndrome (*r* = 0.26, *P* = 0.03).

**Table 3:** Secondary outcomes with their corresponding TPS.

Secondary outcome	TPS1	TPS2	TPS3	*P* value
Low cardiac output syndrome, *n* (%)	1 (5.6)	2 (5.0)	5 (29.4)	0.04
ICU length of stay, day (Mean ± SD)	3.11 ± 0.96	3.42 ± 1.06	4.06 ± 1.14	0.10
Postoperative mechanical ventilation, h (Mean ± SD)	35.17 ± 6.46	33.40 ± 7.43	38.52 ± 5.86	0.06

## Discussion

This study included 75 patients who were followed up for one year after surgery without any deaths. Even post CPB, we use transesophageal echocardiography (TEE) to check for any remaining issues. However, in this study, there were still 17 cases of TPS3. We believe that significant issues indicated by TEE warrant repeat CPB for further correction. Conversely, if the surgeon deems the issue minor, no further corrective action is taken. Four patients received medical intervention within one year after surgery. The study results showed that TPS3 was a risk factor for re-intervention after TOF repair. However, there was no association between TPS and postoperative intensive care unit stay or mechanical ventilation time. Additionally, higher TPS classification was observed in patients who developed low cardiac output syndrome after surgery.

A study included 104 patients who underwent TOF repair showed a one-year rate of freedom from first catheter reintervention of 55% and a rate of freedom from surgical reintervention of 84%, which is significantly higher than the rates observed in our study (11). This difference might be attributed to the fact that the median age and weight of their study population were 11 (4.0–20) days and 2.9 (2.5–3.5) kg, respectively, both significantly lower than in our study. Additionally, 57.4% of their patients were diagnosed with TOF with pulmonary atresia, a condition that was excluded in our study. In another study that included 157 patients undergoing TOF repair with preserved pulmonary valve, 22% of patients belonged to TPS 1, 70% to TPS 2, and 8% to TPS 3 (5). Furthermore, in a study that included 115 pediatric patients under 2 years of age undergoing TOF repair, 25% were classified as TPS 1, 52% as TPS 2, and 23% as TPS 3 (3). However, Lodin et al. found no association between TPS and the need for re-intervention, duration of postoperative monitoring, length of hospital stays, or hospital costs after TOF surgery. This may be due to differences in the study population and surgical strategies employed by the surgeon. The incidence of low cardiac output syndrome after the TOF repair of pulmonary artery preservation surgery is positively correlated with TPS grading, which may be due to an increased likelihood of postoperative low cardiac output syndrome resulting from incomplete correction of cardiac anomalies during surgery. In our study, TPS grading was not associated with postoperative monitoring time or mechanical ventilation time. Nathan et al. conducted a multicenter prospective cohort study to examine the relationship between the RLS score (modified TPS) and early prognosis following the correction of congenital cardiac malformations. Their findings revealed that the RLS score after repairing TOF was not significantly associated with the overall duration of postoperative hospital stay, duration of mechanical ventilation, or length of stay in the postoperative ICU (12).

The TPS for TOF was initially developed in 2007 using data from patients who underwent surgery in 2004 (4). Multiple studies have demonstrated the utility of TPS in predicting early and midterm outcomes, neurodevelopmental outcomes, and healthcare costs following surgical intervention for common congenital heart diseases (5, 6, 8, 12–15). Our study also confirmed that TPS can be used to predict the risk of intervention during the early postoperative period in TOF patients. Although our results lack statistical significance due to the small number of positive cases and the total sample size, they still indicate that TPS3 is a high-risk factor requiring reoperation within one year after TOF surgery.

Although this study is a single-center retrospective study with limitations such as small sample size, missing data, and loss to follow-up, we employed strict inclusion and exclusion criteria to reduce bias from confounding factors known to affect outcomes such as extreme age, emergency presentation, extreme weight, and pulmonary atresia. Therefore, the conclusions drawn from this study are reliable, namely that patients classified as TPS 3 are at higher risk of requiring reintervention within one year after repairing of TOF and should be closely monitored. Furthermore, TPS scoring is based on echocardiography results before discharge and is used to identify high-risk TOF patients postoperatively. If TPS classification based on intraoperative transesophageal echocardiography were available, it could alert surgeons to residual lesions that require further correction during surgery, potentially improving outcomes for TOF patients.

## Conclusion

TPS3 is a risk factor for reintervention within one year after TOF repair highlighting the need for close clinical follow-up of these patients. A higher TPS score was observed in patients who developed LCOS after TOF repair, suggesting the presence of residual heart defects that were not adequately corrected and contributed to the development of low cardiac output syndrome. Therefore, prompt bedside echocardiographic evaluation should be performed to assess the adequacy of heart defect correction when low cardiac output syndrome occurs after surgery.

## Data Availability

The original contributions presented in the study are included in the article/Supplementary Material, further inquiries can be directed to the corresponding author.
